# 

**Published:** 1845-03

**Authors:** 


					﻿Our Journal.—The present number completes the 1st volume
of our Journal. We have to congratulate our friends and our-
selves upon the increasing popularity of our enterprise, evinced
by the demand for the back numbers of our present vol., by the
continual increase of our subscription list, and the complimentary
letters volunteered by many of our subscribers. It is with plea-
sure that we announce to our friends that our Journal is no longer
to be considered an ephemeral work, but as established upon a
firm foundation. Our monthly visits may still be expected. The
first number of our second volume will be issued in April. No
change in the form of the Journal, in the publishers, in the editor,
or in the price of subscription, is anticipated. On our part, we
promise our readers increased exertion, to render our next volume
profitable and interesting; and we flatter ourselves, that our
greater experience will assist our future endeavors, while the in-
crease of our facilities, will, we trust, render our task more easy.
We also have to thank our exchanges for the courtesies which
they have extended to us, and the flattering notices which they
have frequently made of our pages, hoping that our second vol-
ume may be received by them with equal favor.—Ed.
INDEX.
Abscess at the neck of the Bladder, causing retention of Urine. By
Wm. B. Herrick, M.D., Lecturer on Anatomy in Rush Medical
College,......................................................... 105
Address on Insanity. By John Evans, M. D.......................... 30
Alcoholic Odor detected in Serous Effusion in the Ventricles of the
Brain. By L. Bradley, M.D., Elgin, Kane Co., Ills............. 27
American Journal of Insanity,..................................... 95
Ammonia, Cauterization of the Pharynx with....................... 176
Ammonia, Succinate of, a Remedy for Delirium Tremens,.............159
Amputation at the Ankle Joint,................................... 172
Anatomical Atlas by Henry W. Smith,............................... 15
Anchylosis, False, Two Cases of, by D. Brainard, M. D.............. 4
Anemia. By John McLean, Prof. &c., in the Rush Medical Col-
lege.......................................................76,	SI
Anencephalous Foetus, with unusual Malformation of the Heart. By
Daniel Brainard, M. D., Prof., &c............................. 22
Aneurism of the Aorta,........................................... 158
Aneurism of the Femoral Artery,................................... 87
Anomalous Disease of the Tongue and Feet, occurring in Indiana.
By Graham N. Fitch, M. D., Prof., &c., in the Rush Medical
College,.................................................... 165
Antidotes,....................................................... 110
Aorta, Compression of, as a Remedy for Uterine Haemorrhage after
Delivery.	By Daniel	Brainard,	M.	D.,	Prof., &c.,............ 40
Aptlue, Nature and Treatment of...................................120
Articular Rheumatism,	Nature and	Treatment of.................... 156
Atmospheric Air, Exclusion of in certain local diseases. By Marshall
Hall, M.D.,..................................................Ill
Auscultation, Signs of in Young Children,........................ 175
Belladonna as a prophylactic in Scarlatina,......................  11
Black Tongue,..............................................17,	33, 151
Bladder, Abscess in the neck of...................................105
Bougies with Alum,............................................... 180
Boston Medical and Surgical Journal,.............................. 94
Burn—Deformity produced by, successfully treated byPlastic Opera-
tion. By Daniel Brainard, M. D., Prof., &c.................... 57
Calomel, Utility of in Typhoid Fevers,............................ 47
Camphor, Effects of.............................................. 109
Camphor a Preservative of Ergot of Rye,...........................169
Canada Leprosy,.................................................. 169
Chlorosis, Syphiltic.............................................. 171
Chlorosis, Preparations of Iron dangerous in certain forms of..... 61
Cholera Infantum,................................................. 49
Circular to Physicians,...........................................127
Clavicle, Fracture of............................................. 133
Croton Oil Plaster,............................................... 181
Cyclopedia of Practical Medicine,................................. 48
Dedication of the Edifice of the Rush	Medical	College,............160
Delirium Tremens, Succinate of Ammonia a	Remedy	for............159
Dictionary of Practical Medicine, by James Copland, M. D., F.R.S.. 126
Dislocation of the Shoulder. By D. Brainard, M. D., Prof.. &c.___ 42
Dissector, The, by Erasmus Wilson, M.D............................ 15
Drake, Dr. and the Rush Medical College,........................  135
Ergot of Rye, Therapeutic Action and Uses of......................117
Ergot of Rye, Camphor a Preservative of.......................    169
Erysipelas, Epidemic, called Black Tongue,......................17,	33
Erysipelas, Epidemic, as it occured in La Porte Co., Indiana........... 36
Erysipelas, Treatment of with Ointment of Nitrate of silver,.......141
Exchanges, Our.................................................... 176
Fecundation of the Mammiferae, Positive Theory of..................181
Fever, Intermittent, Treatment of................................   65
Fevers, Treatment of by Salines,.................................   12
Forry, Dr. Samuel, Death of......................................  144
Fracture of the Clavicle,..........................................133
Gums. Scarification of during Dentition,...........................107
Human Anatomy, General and Special, by Erasmus Wilson,.............112
Introductory, Editor’s,............................................. 1
Iron, Preparations of, dangerous in certain forms of Chlorosis,....	61
Leprosy, Canada.................................................   169
Loins, Pains of...................................................  92
Mamma, Fibrous Tumors of........................................... 16
Mackintosh’s Practice of Medicine,....................r............ 95
Malpractice, Trial for.............................................142
Medicines in a liquid form, Advantages of.......................... 48
Medical Practice, State Legislation respecting..................... 97
Medical Schools,..............................................32, 80, 95
Mercurial Ointment and Blue Mass, Variation in the strength of.....	60
Mortification, Case of Dry, preceded by Convulsions, in a Puerperal
Patient. By G. N. Fitch, M.D., Prof., &c.......................149
New Orleans Medical Journal,...................................... 142
Norris’ Introductory Lecture, Extract from......................... 62
Ova, Periodical Ripening of, &c....................................181
Parker, Dr., Notice of............................................. 63
Pharynx, Cauterization of	with	Ammonia,..........................176
Piles, Nature and Treatment of.....................................140
Plethora, Treatment of by	Saline	Medicines,.........................168
Principles and Practice of Surgery, by Robert Druitt, Surgeon, &c.. 112
Puerperal Fever,....................................................137
Readers and Correspondents, Notice to.........................32, 64, 96
Report of the Directors and Superintendents of the Ohio Lunatic
Asylum,........................................................ 28
Review of Dr. Caldwell’s pamphlet entitled Physiology Vindicated, by
Robert Peter, M. D., Prof., &c................................. 31
Rickets, Treatment of.............................................. 13
Rubeola, Urtication in the Treatment of............................ 61
Rush Medical College,.......................................64,	135, 160
Rush Medical College, Surgical Clinics,...............129,145, 161, 177
Scarlet Fever, Treatment of......................................... 168
Shivering, a diagnostic in Thoracic Inflammation................... 14
Small Pox, Results of Observations on, in persons who had been vac-
cinated, ..................................................... 173
Spleen, Fatal Case of Rupture of.................................   79
Styptics, Medicinal Mixtures employed as.......................... 170
Sudden Death, Two Cases of, with Autopsical Examinations,..........113
Surgical Patents,.................................................. 16
Sypliiltic Chlorosis,.... ;.....................................   171
Tannin, Pure, The Uses of......................................... 174
Tic Douloureux of the Face and Head................................ 45
Uterus, Rupture of, with occlusion of the Vagina,.................. 25
Visceral Disease, Fatal Case of, with Cerebral Symptoms. By J.
Stickel, M.D...................................................179
Vaccinia, The protective powers of................................ 122
Vesicant, Extemporaneous,........................................   62
Wilson’s Dissector, Notice of...................................... 15
TO SUBSCRBERS.
The Publishers feel themselves obliged to call upon the
subscribers to the Journal to remit the amount of their subscriptions.
Though the subscriptions are required in advance, there are a
large number of names upon the list not yet marked paid. The
price of the Journal is so low that they cannot conceive that this
arises from other cause than neglect. Postmasters have the power
and are always willing to frank remittances. The publishers hope
that they will not be obliged again to issue this notice, or be driven
to the disagreeable alternative of stopping the issue of the Journal
to those who have not paid their subscriptions.
ELLIS & FERGUS. Publishers.
THE ILLINOIS MEDICAL AND SURGICAL JOURNAL
PUBLISHERS’ NOTICE.
The publishers of the Illinois Medical and Surgical Journal,
are happy to announce to their subscribers, that they will continue
its publication as before, issuing the first number of the second
volume in April. They take this opportunity to return their
thanks for the increasing patronage which the Journal has re-
ceived at the hands of the medical public and others, and confi-
dently trust in the merits of the work, to sustain itself and extend
its usefulness.
The Journal will be continued upon the same terms as before,
will be issued monthly, each monthly number containing 16 pages
octavo, with an index and title page in the last number.
It will still be conducted, in the editorial department, by James
V. Z. Blaney, M. D., Professor of Chemistry and Pharmacy, in
the Rush Medical College.
				

